# Circular RNA profile of infantile hemangioma by microarray analysis

**DOI:** 10.1371/journal.pone.0187581

**Published:** 2017-11-02

**Authors:** Cong Fu, Renrong Lv, Guangqi Xu, Linfeng Zhang, Jianhai Bi, Li Lin, Xiaowen Liu, Ran Huo

**Affiliations:** Department of Burn and Plastic Surgery, Shandong Provincial Hospital Affiliated to Shandong University, Jinan, Shandong Province, China; University of Toronto, CANADA

## Abstract

**Background:**

Circular RNAs (circRNAs) are a recently identified class of noncoding RNAs that participate in several physiological processes. However, the expression of circRNAs in infantile hemangioma (IH) remains unknown.

**Methods:**

The profile of circRNAs was assessed by microarray in four pairs of IH and adjacent skin tissues. The expression of circRNAs was validated by quantitative reverse transcription polymerase chain reaction (qRT-PCR). Furthermore, circRNA-microRNAs (miRNA)-mRNA networks were constructed using bioinformatics tools.

**Results:**

234 up- and 374 down- regulated circRNAs were identified in IH by microarray. Among them, the expression of two up-regulated circRNAs (hsa_circRNA_100933 and hsa_circRNA_100709) and one down-regulated circRNA (hsa_circRNA_104310) was confirmed by qRT-PCR. In addition, 3,019 miRNA response elements (MREs) of circRNAs were predicted, and two circRNA-miRNA-mRNA networks were constructed, including 100 and 94 target genes of hsa_circRNA_100933 and hsa_circRNA_104310, respectively. GO and pathway analysis showed that both networks participated in angiogenesis and vascular development-related biological processes.

**Conclusions:**

This is the first study to reveal the profiling of circRNAs in IH and pave the way for further characterization of the role of circRNAs in the pathogenesis of IH.

## Introduction

Infantile hemangioma (IH) is a common vascular tumor in infancy, especially in females and premature with an increasing incidence of 3–10%[1]. Most IHs possess a unique life cycle composed of proliferation and involution stage; the proliferation is usually rapidly in the first year, followed by spontaneous resolution until 3–7 year old [2]. Notably, the rapid growth during proliferation stage may lead to ulceration, scarring, disfiguring, or even life-threatening complications [3].

Angiogenesis and vasculogenesis have been recognized as underlying neovascularization in IH [4,5]. The histopathological feature of proliferative IH includes masses of endothelial cells and closely packed capillaries. A high level of circulating endothelial progenitor cells (EPCs) support the hypothesis that IHs originate from intrinsic EPC [6,7]. Moreover, the theory of placenta origin suggests that the impairment of placenta during gestation may contribute to endothelial embolization and proliferation [8]. Accumulating evidence suggests that the placenta antigen glucose transporter 1 (GLUT 1) expresses in endothelial cells of IH and is an immunochemical marker for IH [9]. The extrinsic factors, such as hypoxia and acidosis and intrinsic factors, such as hypoxia-inducible factor 1 alpha (HIF1-α), vascular endothelial growth factors (VEGFs), and other angiogenic factors have been suggested to contribute to the development of IH [10,11]. Despite multiple hypotheses, the pathogenesis of IH is largely unknown.

Based on RNA sequencing data, a majority of human transcripts are non-coding RNAs (ncRNAs), with < 2% of the genome encoding the proteins [12]. An increasing volume of evidence suggests that ncRNAs are vital regulators in the vascular development of various disorders. Our previous study comparing the profile of long noncoding RNAs (lncRNAs) in IH and adjacent normal tissues revealed that lncRNAs are involved in angiogenesis and vascular biology [13]. Moreover, microRNAs (miRNAs), known as the common group of small ncRNAs, have been proved to play a pivotal role in IH pathogenesis. A recent study showed that chromosome 19 miRNA cluster (C19MC) was overexpressed in both IH tissues and blood samples, identifying C19MC as a candidate biomarker of IH [14]. MiR-382 is overexpressed in IH endothelial cells and contributes to IH development [15]. The inhibition of miR-130a suppresses the cell growth and angiogenesis of IH [16]. Nonetheless, the mechanism underlying the regulation of miRNA in IH necessitates further studies.

Circular RNAs (circRNAs) are a recently identified class of ncRNAs that participate in several physiological processes[17]. Although circRNAs were discovered in 1972, they were mistaken for derivatives of mis-splicing [18,19]. The biology and function of circRNAs have aroused attention until 2012 thanks to high throughput technology[20]. Importantly, circRNAs have been demonstrated to serve as miRNA sponges for regulating gene expression [21,22]. Some circRNAs compete with mRNAs for the same miRNA binding sites to form an integral network of posttranscriptional regulation, termed as the competing endogenous RNAs (ceRNAs) network [23,24]. Recently, circRNAs profiling have been identified in several kinds of disorders, especially in tumors, such as cutaneous squamous cell carcinoma[25], epithelial ovarian carcinoma[26], basal cell carcinoma[27]. However, the circRNAs involved in IH are yet to be explored. Interestingly, a recent study identified a circRNA profile in human umbilical endothelial cells under hypoxic or normoxic conditions and indicated that circRNA cZNF292 was up-regulated by hypoxia and controlled the formation of tubers and spheroid sprouting of endothelial cells [28]. These characteristics indicate that circRNAs might play an important role in the etiology of IH.

Therefore, in this study we identified the profile of circRNAs in four pairs of proliferative IH and adjacent skin tissues by high-throughput microarray. Subsequently, a total of 8 circRNAs were examined using quantitative reverse transcription polymerase chain reaction (qRT-PCR) in 10 paired IH and adjacent skin tissues. The most significant up- (hsa_circRNA_100933) and down-regulated (hsa_circRNA_104310) circRNA were chosen for further study. Next, we predicted the interaction between circRNAs and miRNAs and constructed hsa_circRNA_100933-miRNA-mRNA and hsa_circRNA_104310-miRNA-mRNA networks using the bioinformatics approach.

## Methods

### Ethics statement

This study was approved by the Institutional Ethics Review Board of Shandong Provincial Hospital Affiliated to Shandong University (No. 2016–206). Written informed consents were obtained from the guardians on behalf of the children enrolled in this study.

### Patients and samples

The proliferative IH was diagnosed by two independent doctors according to clinical and pathological features. IH samples (Table 1) were excised at the Department of Plastic Surgery of Shandong Provincial Hospital between June 2016 and February 2017. Four pairs of samples were used for microarray. Then, total 10 pairs were used for further validation. The excised samples were immersed overnight in RNAlater (Qiagen, Hilden, Germany) at 4°C and preserved at -80°C.

**Table 1:** Clinicopathological characteristics of IH patients.

Subject	Gender	Age	Localization	Previous therapy	Immunochemistry
1	Female	5 months	Thoracic wall	None	GLUT+
2	Female	4 months	Back	None	GLUT+
3	Male	4 months	Back	None	GLUT+
4	Female	7 months	Back	None	GLUT+
5	Female	6 months	Lumber region	None	GLUT+
6	Female	4 months	Back	None	GLUT+
7	Male	6 months	Scalp	None	GLUT+
8	Female	3 months	Thoracic wall	None	GLUT+
9	Female	9 months	Back	None	GLUT+
10	Male	2 months	Back	None	GLUT+

### RNA isolation and quality control

RNA from each sample was isolated by TRIzol reagent (Invitrogen life technologies, Carlsbad, USA) based on the manufacturer's instructions. The purity and concentrations of total RNA samples were determined with NanoDrop ND-1000. RNA integrity was tested by denaturing agarose gel electrophoresis.

### Microarray and data analysis

The complete microarray data were deposited in Gene Expression Omnibus (GEO) database (accession number GSE93522 [NCBI tracking system #18429863]). Sample labeling and array hybridization were performed based on the Arraystar’s protocol. Total RNA from each sample was treated with RNase R (Epicenter, Madison, USA) to enrich circular RNAs and remove linear RNAs. Subsequently, fluorescent cRNA were obtained by amplification and transcription of circular RNAs through random priming (Arraystar Super RNA Labeling Kit). The labeled cRNAs (pmol Cy3/μg cRNA) were purified by RNeasy Mini Kit (Qiagen) and assessed with NanoDrop ND-1000. Then blocking agent (10× 5uL) and fragmentation buffer (25× 1uL) were added to make each labeled cRNA fragmented, followed by heating at 60°C for 30 min. Labeled cRNA was diluted with hybridization buffer (2× 25uL). Hybridization solution (50 uL) was added into the gasket slide. Finally, microarray slide was incubated for 17 h at 65°C in an Agilent Hybridization Oven. The hybridized arrays were washed, fixed, and scanned using the Agilent Scanner G2505C.

R software limma package was used for data normalization and calculation. The fold-change between the IH and adjacent skin tissues was calculated. The statistical significance was calculated by t-test. circRNAs with fold-change ≥2and P-values≤0.05 were regarded as a significant differential expression.

### qRT-PCR validation

Total RNAs of 10 paired IH and adjacent skin tissues were reverse transcribed to synthesize cDNA by Gene Amp PCR System 9700 (Applied Biosystems, Carlsbad, USA). The selected circRNAs should meet the criteria that fold change was more than 4 and raw intensity of each sample was greater than 100. In addition, circRNAs with miRNA response elements (MREs) related to tumor progression and angiogenesis reported in the literatures were selected preferentially. Finally, 8 circRNAs were selected, including 5 up-regulated circRNAs (circRNA_100709, circRNA_102116, circRNA_051239, cricRNA_100933 and circRNA_102039) and three down-regulated circRNAs (circRNA_023016, circRNA_001654 and circRNA_104310), for amplification by specific divergent primers (S1 Table). The housekeeping gene *Human β-actin* was selected as an internal reference. All cDNAs were assembled in the ViiA 7 real-time PCR System (Applied Biosystems) in triplicates each. The reaction was based on the following procedures: 95°C, 10 min; 40 PCR cycles (95°C, 10 s; 60°C, 1 min). The single-peak appearance of the melt curve demonstrated the specificity of PCR primers. The relative level of circRNAs was computed by the 2^-ΔΔCt^ method.

### Construction of circRNA-miRNA-mRNA networks

The miRNA response elements (MREs) of circRNAs was predicted using Arraystar's miRNA target prediction software based on TargetScan [29] and miRanda [30]. The interaction of circRNAs and miRNAs were displayed by Cytoscape 3.01. The target genes of circRNAs were obtained by the integration of miRWalk 2.0 (miRWalk, miRanda, PITA, Targetscan) [31] and mRNA profile (GSE78811) previously [13]. GO analysis was used to predict the potential functions of the target genes by the Database for Annotation, Visualization, and Integrated Discovery (DAVID) online tool [32,33], including the categories of biological process (BP), molecular function (MF) and cellular component (CC). KEGG Ontology-Based Annotation System (KOBAS) 2.0 was used to proceed the pathway analysis of target genes, which incorporates five pathway databases [KEGG Pathway, PID, BioCyc, Reactome, and Panther] [34].

### Statistical analysis

All data were presented as the mean ±standard error of mean (SEM). Student’s t-test (two-tailed) was used for data analysis. GraphPad Prism 5 and Microsoft Office were utilized for other statistics. P < 0.05 was considered to be statistically significant.

## Results

### Analysis of differentially expressed circRNAs

**Fig 1:**
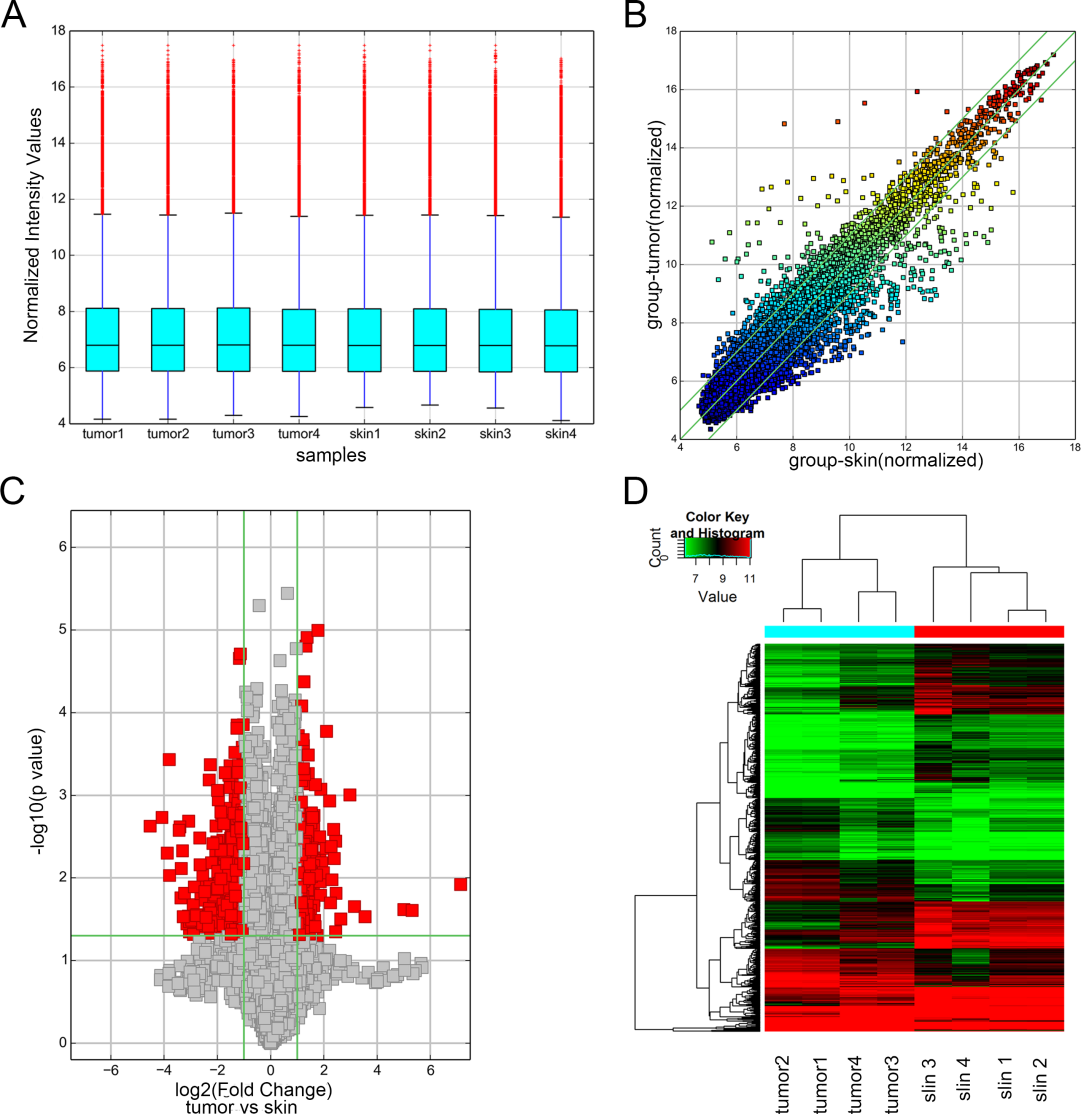
Differential expression of circRNAs between IH and adjacent skin tissues by microarray. (A) Box plot showed that all the 8 samples were normalized. (B) Scatter-Plot was used for assessing the variation of circRNA expression between IH and adjacent skin tissues. The circRNAs above the top and below the bottom green lines indicated > 2.0-fold change in circRNAs between the two groups. (C) Volcano Plots were displayed for visualizing the differential expression of circRNAs. The red points in the plot represent the differentially expressed circRNAs with statistical significance. (D) Hierarchical cluster analysis of all the deregulated circRNAs.

**Fig 2:**
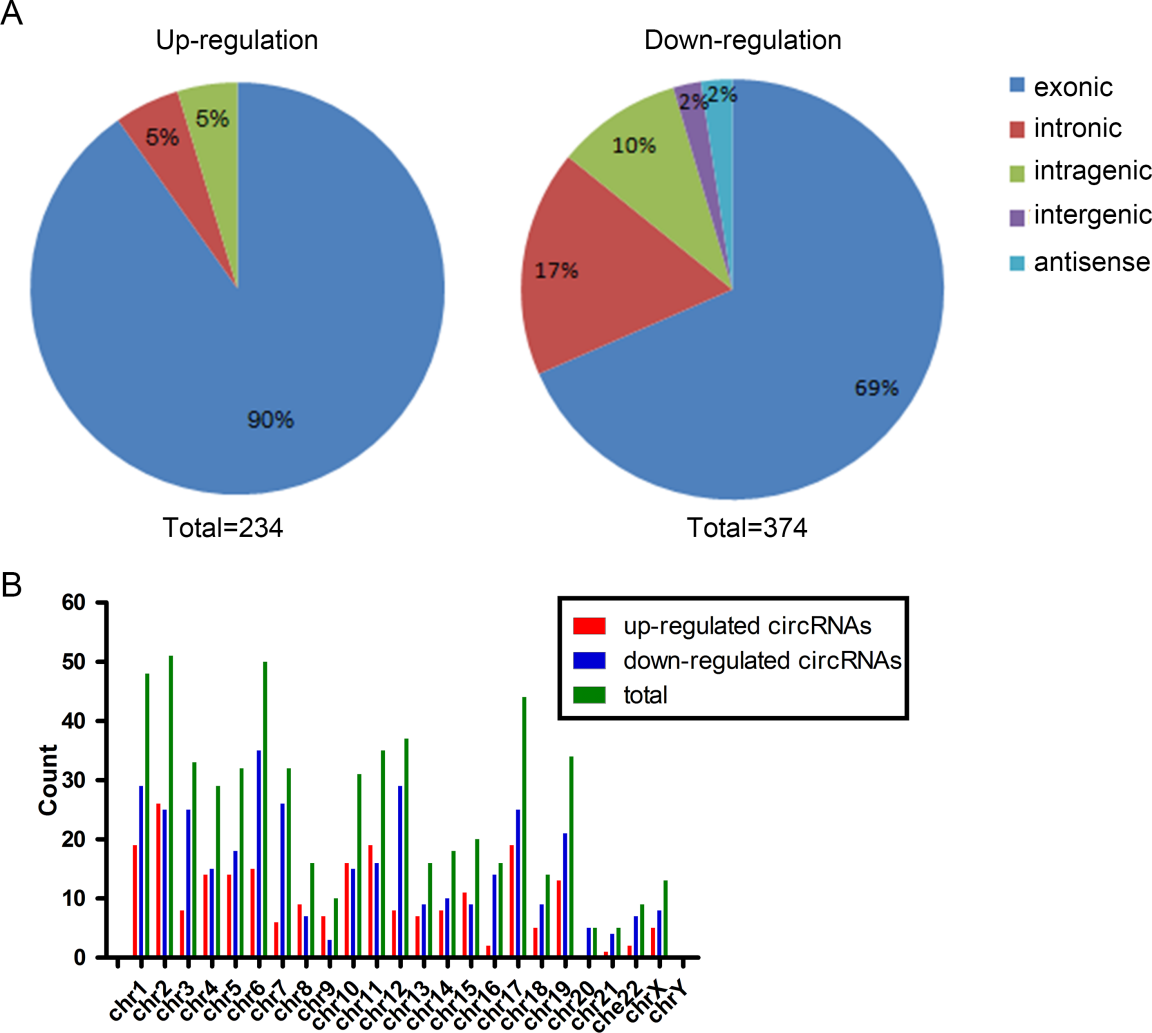
Characteristics of differentially expressed circRNAs. (A) Classification of deregulated circRNAs. (B) Distribution of circRNAs on human chromosomes.

In total, 13617 circRNAs were analyzed by microarray in 4 pairs of IH and adjacent skin tissues (S2 Table). Box plot demonstrated the intensity of all the samples after normalization (Fig 1A). Differentially expressed circRNAs between IH and adjacent skin tissues were acquired based on fold change filtering (Fig 1B). Finally, 234 up- and 374 down-regulated circRNAs (S3 Table) were identified by fold change ≥ 2.0 and P-value < 0.05 (Fig 1C). Hierarchical clustering was performed to visualize the differential circRNAs (Fig 1D). The top 25 up- and down-regulated circRNAs were displayed in Tables 2 and 3. The classification schematic revealed that up-regulated circRNAs included 211 exonic, 12 intronic, and 11 intragenic, while the down-regulated circRNAs included 256 exonic, 65 intronic, 36 intragenic, 8 intergenic, and 9 antisense (Fig 2A). In addition, we summarized the localization of circRNAs on human chromosomes (Fig 2B). The data showed that the expression of circRNA in IH is much different from that of adjacent skin tissues.

**Table 2:** Top 25 up-regulated circRNAs in IH.

circRNA	P-value	FDR	FC	chrom	strand	type	Gene Symbol
hsa_circRNA_100709	0.012	0.131	140.473	chr10	-	exonic	FAM53B
hsa_circRNA_102116	0.025	0.168	39.712	chr17	-	exonic	ZNF652
hsa_circRNA_051239	0.024	0.166	32.026	chr19	-	exonic	ATP5SL
hsa_circRNA_051238	0.029	0.179	11.627	chr19	-	exonic	ATP5SL
hsa_circRNA_101214	0.022	0.161	8.862	chr12	+	exonic	GPR133
hsa_circRNA_100933	0.001	0.070	7.844	chr11	-	exonic	NOX4
hsa_circRNA_102538	0.031	0.182	6.174	chr19	+	exonic	CAPNS1
hsa_circRNA_401782	0.015	0.143	5.473	chr17	+	exonic	TAF15
hsa_circRNA_101439	0.004	0.093	5.430	chr14	-	exonic	WARS
hsa_circRNA_101985	0.045	0.209	5.426	chr17	+	exonic	MAP2K4
hsa_circRNA_100945	0.005	0.107	5.218	chr11	-	exonic	GUCY1A2
hsa_circRNA_104162	0.003	0.088	5.128	chr6	-	exonic	HACE1
hsa_circRNA_403382	0.010	0.126	4.192	chr5	+	exonic	RASGRF2
hsa_circRNA_102039	0.004	0.097	4.814	chr17	+	exonic	TAF15
hsa_circRNA_023983	0.020	0.156	4.728	chr11	-	exonic	NOX4
hsa_circRNA_003030	0.001	0.073	4.640	chr10	+	exonic	PRKG1
hsa_circRNA_402294	0.000	0.048	4.265	chr2	+	exonic	ACTR2
hsa_circRNA_001992	0.004	0.098	4.190	chrX	-	exonic	FIRRE
hsa_circRNA_104163	0.008	0.118	3.929	chr6	-	exonic	HACE1
hsa_circRNA_016121	0.009	0.121	3.822	chr1	+	exonic	SOX13
hsa_circRNA_007104	0.008	0.109	3.772	chr12	-	exonic	DPY19L2
hsa_circRNA_101996	0.001	0.068	3.674	chr17	+	exonic	SPECC1
hsa_circRNA_103837	0.016	0.145	3.647	chr5	+	exonic	ITGA1
hsa_circRNA_102881	0.011	0.127	3.629	chr2	-	exonic	HECW2
hsa_circRNA_404446	0.008	0.106	3.589	chr1	-	exonic	CAPZB

FDR: False discovery rate, FC: Fold change

**Table 3:** Top 25 down-regulated circRNAs in IH.

circRNA	P-value	FDR	FC	chrom	strand	type	Gene Symbol
hsa_circRNA_000881	0.002	0.087	23.090	chr10	+	intronic	MSI2
hsa_circRNA_023016	0.002	0.079	16.953	chr17	+	exonic	RBM4
hsa_circRNA_001654	0.005	0.102	14.761	chr11	+	intronic	CNPY3
hsa_circRNA_405814	0.000	0.056	14.024	chr6	+	intronic	ZNF470
hsa_circRNA_101491	0.009	0.121	13.924	chr19	+	exonic	MAPKBP1
hsa_circRNA_406655	0.002	0.088	10.866	chr15	-	intronic	CTC-228N24.3
hsa_circRNA_406698	0.002	0.087	10.626	chr5	+	Intergenic	*
hsa_circRNA_001490	0.017	0.148	10.318	chr5	+	exonic	KIF2A
hsa_circRNA_001409	0.007	0.115	10.247	chr5	+	intronic	DGCR8
hsa_circRNA_001653	0.004	0.098	9.935	chr22	+	intronic	DUSP22
hsa_circRNA_104310	0.029	0.178	9.730	chr6	+	exonic	ZDHHC4
hsa_circRNA_103801	0.015	0.142	9.427	chr7	-	exonic	MYO10
hsa_circRNA_406157	0/035	0.189	8.828	chr5	+	Intragenic	AGPAT3
hsa_circRNA_103372	0.028	0.177	8.561	chr21	-	exonic	IP6K2
hsa_circRNA_002086	0.002	0.083	8.450	chr3	-	exonic	LOC401320
hsa_circRNA_081481	0.044	0.209	8.296	chr7	+	exonic	FBXO24
hsa_circRNA_406768	0.033	0.185	8.274	chr7	-	exonic	MDGA1
hsa_circRNA_025522	0.032	0.183	7.817	chr6	-	exonic	ARHGDIB
hsa_circRNA_102445	0.021	0.158	7.562	chr19	+	exonic	CARM1
hsa_circRNA_405963	0.048	0.216	7.417	chr2	+	intronic	UGGT1
hsa_circRNA_001459	0.012	0.134	7.281	chr11	-	intronic	SPI1
hsa_circRNA_092418	0.028	0.177	6.929	chr15	+	intronic	B2M
hsa_circRNA_101522	0.027	0.175	6.874	chr15	-	exonic	DMXL2
hsa_circRNA_103361	0.038	0.194	6.434	chr3	-	exonic	SMARCC1
hsa_circRNA_103269	0.034	0.187	6.387	chr22	-	exonic	LMF2

FDR: False discovery rate, FC: Fold change

*circRNA located outside of the known gene locus

### qRT-PCR verification of selected circRNAs

**Fig 3:**
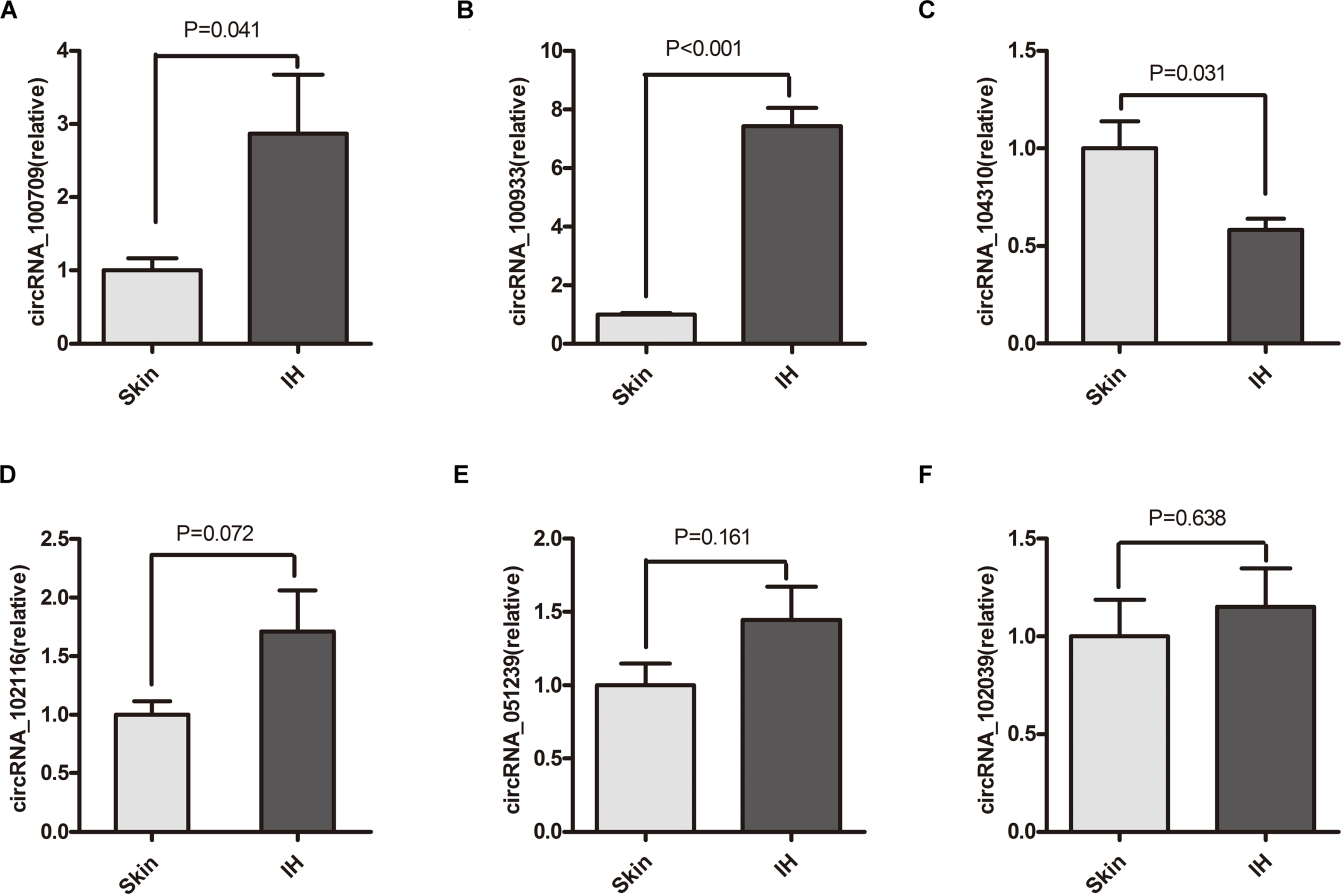
Quantitative real-time PCR validation of selected circRNAs. The relative expression of selected circRNAs (circRNA_100709, circRNA_100933, circRNA_104310, circRNA_102116, circRNA_051239 and circRNA_102039) in IH as compared to paired skin samples.

On the basis of microarray raw intensity and downstream microRNAs, we selected 8 circRNAs from the most significant circRNAs for further verification. The expression of selected circRNAs was substantiated by qRT-PCR in 10 pairs of IH and adjacent skin tissues including the previous microarray group. As a result, six of them (circRNA_100709, circRNA_102116, circRNA_051239, circRNA_100933, circRNA_102039, and circRNA_104310) had the same change direction with microarray results (Fig 3). The other two circRNAs (circRNA_023016 and circRNA_001654) showed a similar expression level between IH and skin tissues. Finally, two up-regulated circRNAs (circRNA_100933 and circRNA_100709) and one down-regulated circRNA (circRNA_104310) were validated with statistical significance.

### Construction of circRNA-miRNA-mRNA network

**Fig 4:**
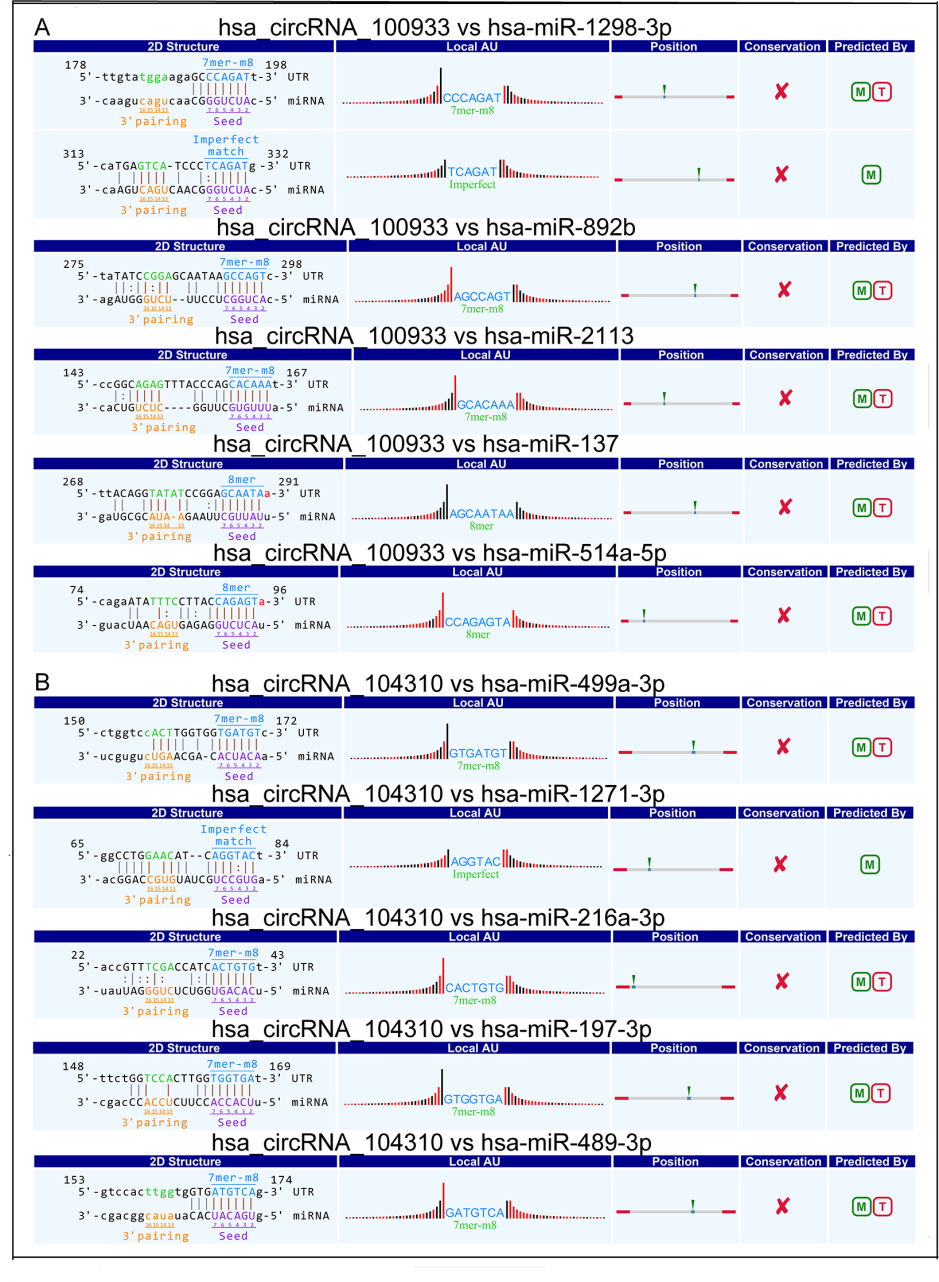
A snippet of detailed annotation for circRNA/miRNA interaction. (A) has_circRNA_100933. (B) has_circRNA_104310.

**Fig 5:**
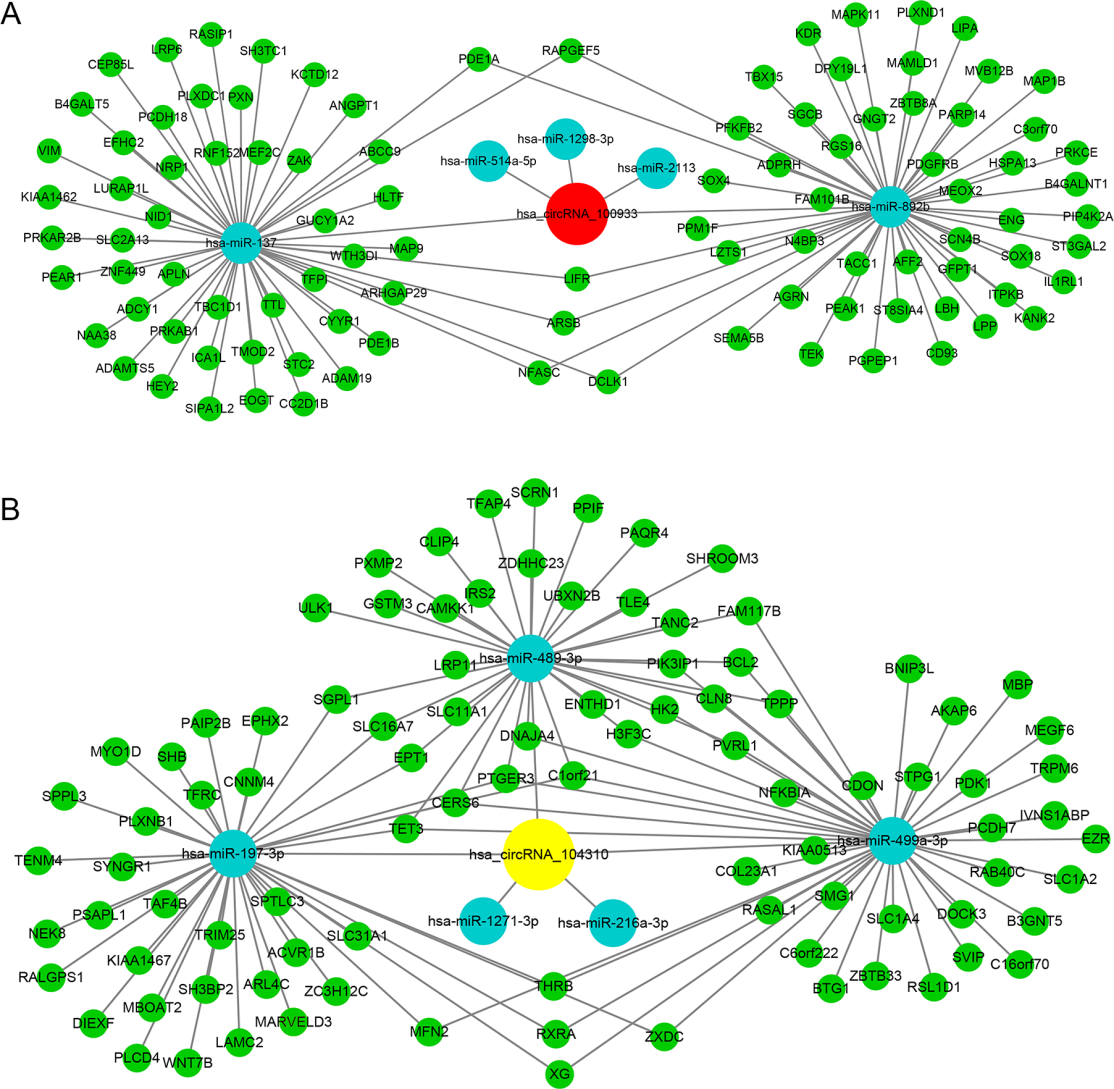
Networks of circRNA-miRNA-mRNA in IH. (A) Hsa_circRNA_100933 with 5 MREs and 94 target genes. (B) Hsa_circRNA_104310 with 5 MREs and 100 target genes. Red represents up-regulation in IH, yellow represents down-regulation in IH, blue represents miRNAs, green represents mRNAs.

According to PCR results, the most significant up- (circRNA_100933, p<0.001) and down-regulated circRNA (circRNA_104310, p<0.05) were selected for further study. In order to explore the functions of circRNAs, we constructed the circRNA-miRNA-mRNA networks based on bioinformatics and the mRNA profile (GSE78811) published by our group previously. Firstly, MREs of circRNAs were predicted by Arraystar’s miRNA target prediction software. In total, 3019 MREs of circRNAs were detected (S3 Table). The interaction between circRNAs and their MREs were represented by Cytoscape (S1 Fig). The potential MREs of hsa_circRNA_100933 were miR-1298-3p, miR-892b, miR-2113, and miR-137, miR-514a-5p (Fig 4A). Moreover, the potential MREs of hsa_circRNA_104310 included miR-499a-3p, miR-1271-3p, miR-216a-3p, miR-197-3p, and miR-489-3p (Fig 4B). Then, 100 and 94 target genes of hsa_circRNA_100933 and hsa_circRNA_104310 were acquired by integration of the target gene prediction and mRNA profile (GSE78811), respectively. The hsa_circRNA_100933/hsa_circRNA_104310-miRNA-mRNA networks were illustrated using Cytoscape (Fig 5).

### GO and pathway analysis of target genes

**Fig 6:**
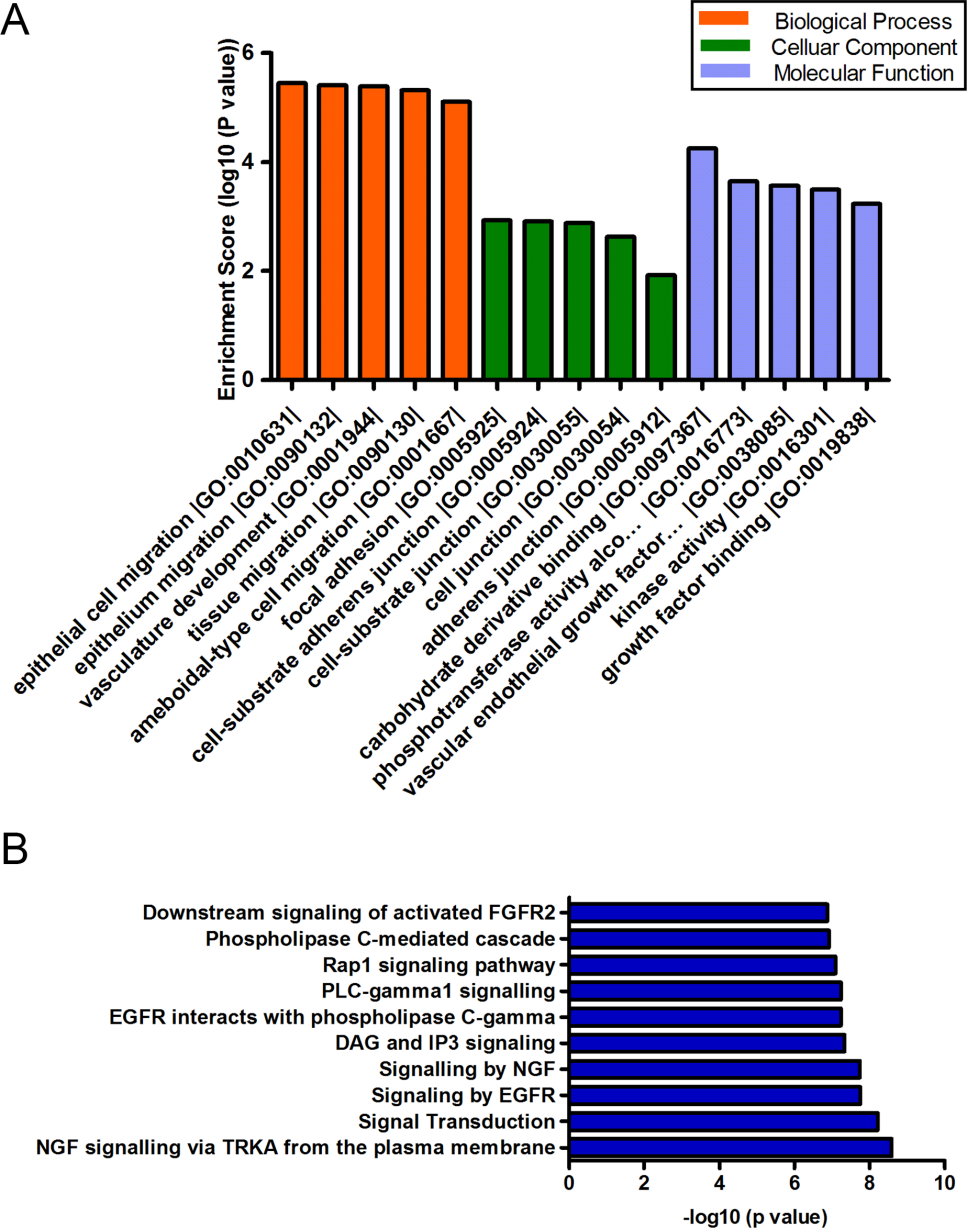
GO (A) and pathway analysis (B) of hsa_circRNA_100933 targeted genes.

**Fig 7:**
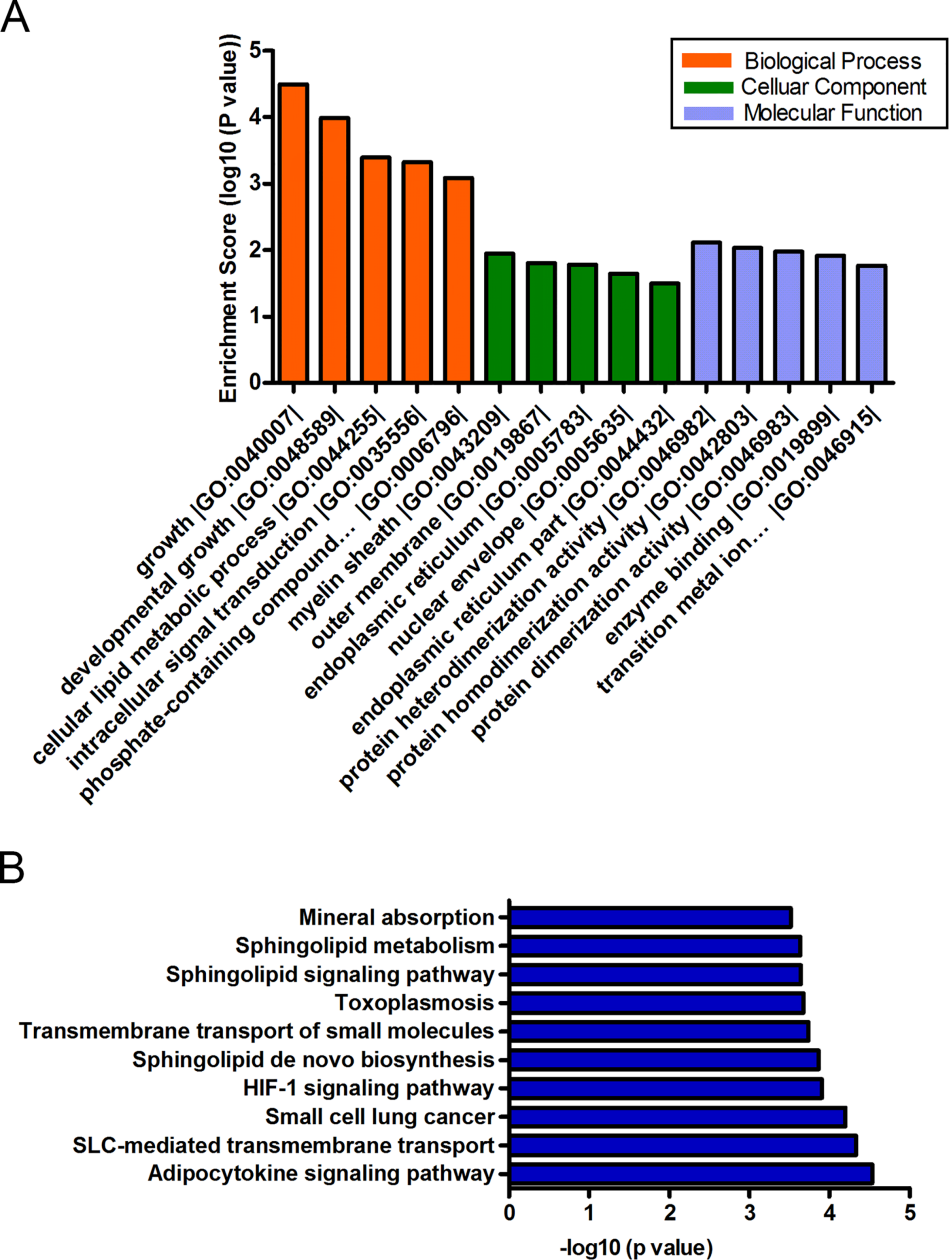
GO (A) and pathway analysis (B) of hsa_circRNA_104310 targeted genes.

To gain further insight into the function of target genes, GO and pathway analysis was conducted using DAVID and KOBAS. The top five significant GO terms of each subgroup (BP, CC, MF) were displayed. Intriguingly, the hsa_circRNA_100933-targeted genes were primarily involved in the physiological processes including epithelial cell migration, epithelium migration, vasculature development, and tissue migration (Fig 6A). The top ten pathway terms were also displayed (Fig 6B), which encompassed several angiogenesis-related pathways, such as signaling by EGFR; EGFR interacts with phospholipase C-gamma and Rap1 signaling pathway. In addition, the significant GO terms of hsa_circRNA_104310 included growth, developmental growth, cellular lipid metabolic processes, and intracellular signal transduction (Fig 7A). Moreover, the HIF signaling pathway was among the top ten pathways of hsa_circRNA_104310 (Fig 7B) that played a vital role in the pathogenesis of IH.

## Discussion

The present study, for the first time, examined the expression of circRNAs in IH and identified 234 up- and 374 down-regulated circRNAs. A majority of the deregulated circRNAs were exonic; the intronic type was more regular in down-regulated circRNAs than those who are up-regulated. In addition, the expression of hsa_circRNA_100933, hsa_circRNA_100709 and hsa_circRNA_104310 was confirmed by PCR. CircRNAs are a large group of stable ncRNAs, which are widely found in various cells and exhibit a critical regulatory role in gene expression [35]. In 2013, Memczak et al. [36] successfully identified more than 1980 kinds of circRNAs using high-throughput technology. Since then, the function of circRNAs in the tumor formation was gradually recognized. The characteristics of circRNAs include high stability, wide variety, transcription from exons, rich MREs, sequence specificity, and conserved across species [37]. These features indicate that circRNAs play a critical role in both transcriptional and post-transcriptional levels, and may serve as a new diagnostic and therapeutic target of IH.

The functional study of circRNAs, which designates them to act as miRNA sponges, is an emerging research area of physiological processes and tumor formation. The common circRNA, CDR1as/ciRS-7, which harbors 71 miR-7 binding sites [38], was shown as the inhibitor of miR-7 in embryonic zebrafish midbrain [36] and islet cells [39,40]. The present study predicted the interaction of circRNAs and miRNAs. Among them, miR-137 targeted by circRNA_100933, has been reported to act as tumor suppressor in various tumors [41,42]. Previous study demonstrated that miR-216a-3p, one of the target miRNAs of circRNA_104310, inhibited gastric and colorectal cancer cell proliferation and migration [43,44]. Moreover, hsa-520d-3p and hsa-miR-520a-5p targeted by hsa_circRNA_001654 and hsa_miR-518f-3p targeted by hsa_circRNA_102116 were implicated as biomarkers in IH [14], suggesting the potency of circRNAs as biomarker candidates in IH.

As circRNA and mRNA can compete for the same miRNA binding sites according to ceRNA theory [45], we constructed two circRNA-miRNA-mRNA networks with an integration of miRWalk prediction and mRNA profile(GSE78811) to gain further insight into the function of circRNAs in IH. As a result, 100 target genes of hsa_circRNA_100933 and 94 target genes of hsa_circRNA_104310 were acquired. Moreover, GO and pathway analysis suggested a number of angiogenesis and vascular development-related physiological processes and pathways. The hsa_circRNA_100933-targeted genes were primarily involved in neovascularization-related biological processes including epithelial cell migration, epithelium migration, vasculature development, and tissue migration. EGFR signaling and Rap1 signaling pathways that included the top 10 pathway terms, played a major role in vasculogenesis and angiogenesis of several vascular diseases [46,47]. Among the top 10 pathways of hsa_circRNA_104310-targeted genes, HIF-1 signaling pathway has been demonstrated as an essential pathogenic mechanism in IH, which could regulate angiogenesis via VEGF signaling [48]. The bioinformatics data indicated that circRNAs may be involved in molecular mechanisms underlying neovascularization and may play a vital role in the mechanism of IH. Nevertheless, the function of circRNAs and the circRNA-miRNA-mRNA network in IH requires further investigations.

## Conclusion

This is the first study to prolife circRNAs expression in IH. Furthermore, the circRNA/miRNA interaction was predicted, and circRNA-miRNA-mRNA networks were constructed using bioinformatics tools. The GO and pathway analysis identified many vasculogenesis- and angiogenesis-related pathways, which may provide new insights into the pathogenesis of IH. However, the function of circRNAs in IH requires further in vitro and in vivo studies.

## Supporting information

S1 Table qRT-PCR primers of circRNAs. (DOCX)

S2 Table CircRNA expression profiling data. (XLS)

S3 Table Differentially-expressed circRNAs. (XLS)

S1 Fig Interaction between circRNAs and miRNAs. (A). Deregulated circRNAs and their MREs, including 234 up regulated circRNAs (red nodes), 374 down deregulated circRNAs (yellow nodes) and MREs (blue nodes). (B). The most significant differentially expressed circRNAs and MREs were enlarged.
(TIF)
