# Supplementary material for: Circular RNA profile of infantile hemangioma by microarray analysis
Source: PLoS One. 2017 Nov 2;12(11):e0187581. doi: 10.1371/journal.pone.0187581 (PMC5667857; doi:10.1371/journal.pone.0187581)
Supplement: S1 Table — (DOCX) [file pone.0187581.s001.docx]

S1 Table qRT-PCR primers of circRNAs

|  | Primer Sequence | Annealing (℃) | PS (bp) |
| --- | --- | --- | --- |
| β-actin（H） | F:5' GTGGCCGAGGACTTTGATTG3'  R :5’ CCTGTAACAACGCATCTCATATT3’ | 60 | 73 |
| hsa_circRNA_102116 | F:5’ TGGTGTGGCAAGGATTTCAAC3'  R:5’ TTCGCATGTAAATGGCATGTCT3’ | 60 | 93 |
| hsa_circRNA_100933 | F:5’ AATCATCCATTTACCCTCACA3'  R:5’ AGAGCTGGTTCGGTTAAGACT3’ | 60 | 97 |
| hsa_circRNA_102039 | F:5’ CTACAGCCACCACACACAAGT3'  R:5’ GTCCATAAGAGGAATCAGTCGT3’ | 60 | 115 |
| hsa_circRNA_104310 | F:5’ GGACACGGTCTTTCTTATTCAGG3'  R:5’ ACAGTGATGGTCGAAACGGTG3’ | 60 | 63 |
| hsa_circRNA_023016 | F:5’ TAAATGGTTTTTCAACTTAGGG3'  R:5’ GAAGGGTGTATTCTCATGCAC3’ | 60 | 165 |
| hsa_circRNA_001654 | F:5’ ATATGTGCAGGGGTTGTGG 3’  R:5’ TCACACCAGCAGACACACTAAG 3’ | 60 | 87 |
| hsa_circRNA_051239 | F:5’ TACCTCCAAGGAGCTAATGG3’  R:5’ CTAAGACCAAATCCCACATCC 3’ | 60 | 91 |
| hsa_circRNA_100709 | F:5’ AAGATGGCACAGCACACGC 3’  R:5’ CTGTCATTTTCCATAATTCCACA 3’ | 60 | 77 |
