# Supplementary figures and images for: Circular RNA profile of infantile hemangioma by microarray analysis
Source: PLoS One. 2017 Nov 2;12(11):e0187581. doi: 10.1371/journal.pone.0187581 (PMC5667857; doi:10.1371/journal.pone.0187581)

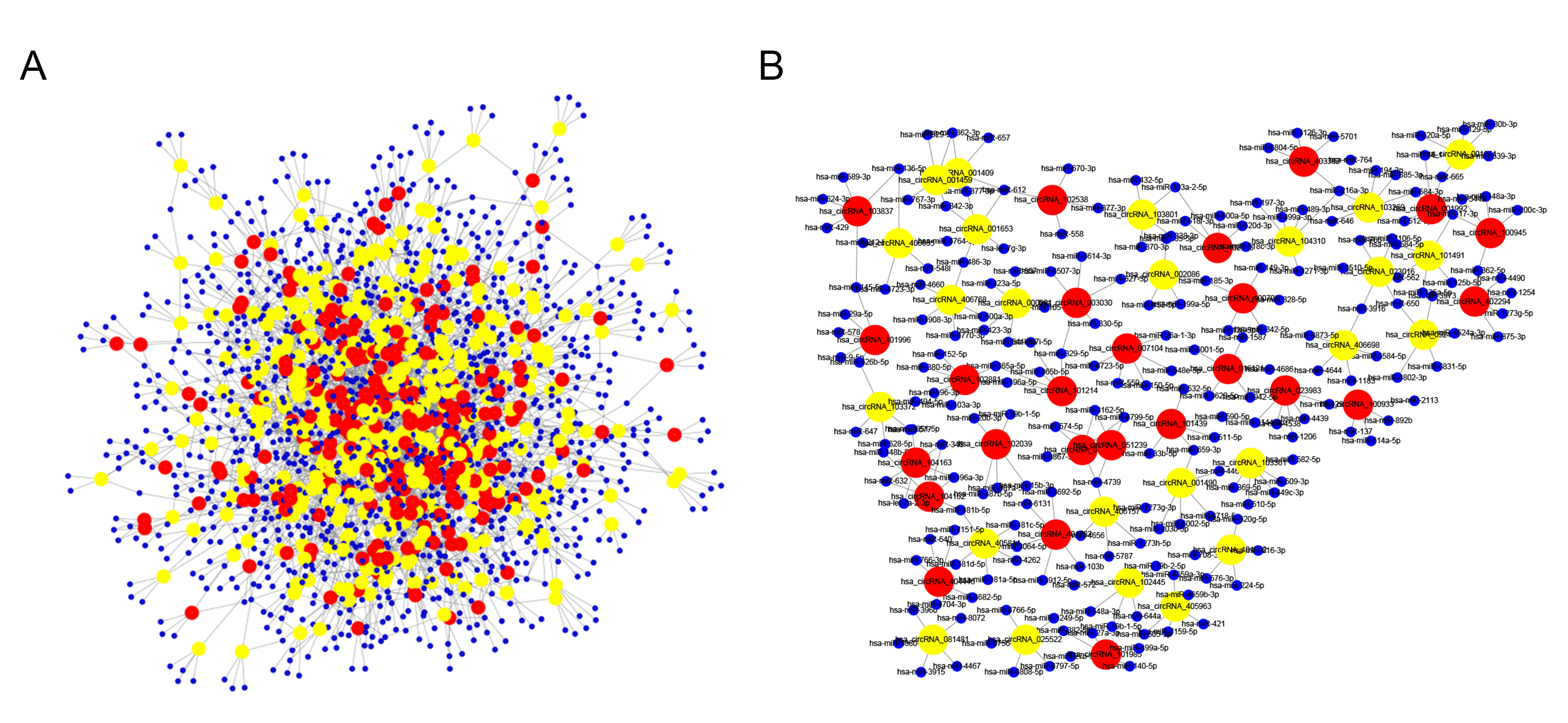

Supplement: S1 Fig — (A). Deregulated circRNAs and their MREs, including 234 up regulated circRNAs (red nodes), 374 down deregulated circRNAs (yellow nodes) and MREs (blue nodes). (B). The most significant differentially expressed circRNAs and MREs were enlarged. (TIF) [file pone.0187581.s004.tif]
